# NAT10/ac4C/FOXP1 Promotes Malignant Progression and Facilitates Immunosuppression by Reprogramming Glycolytic Metabolism in Cervical Cancer

**DOI:** 10.1002/advs.202302705

**Published:** 2023-10-11

**Authors:** Xiaona Chen, Yi Hao, Yong Liu, Sheng Zhong, Yuehua You, Keyi Ao, Tuotuo Chong, Xiaomin Luo, Minuo Yin, Ming Ye, Hui He, Anwei Lu, Jianjun Chen, Xin Li, Jian Zhang, Xia Guo

**Affiliations:** ^1^ Shenzhen Key Laboratory of Viral Oncology; Ministry of Science and Innovation Shenzhen Hospital Southern Medical University Shenzhen Guangdong China; ^2^ The Third School of Clinical Medicine Southern Medical University Guangzhou Guangdong China; ^3^ Department of Ultrasound South China Hospital of Shenzhen University Shenzhen Guangdong China; ^4^ Department of Critical Care Medicine Shenzhen Hospital, Southern Medical University Shenzhen Guangdong China; ^5^ Department of Stomatology Longhua People's Hospital Affiliated with Southern Medical University Shenzhen Guangdong China; ^6^ School of Stomatology Southern Medical University Guangzhou Guangdong China; ^7^ Department of Obstetrics and Gynecology Shenzhen Hospital of Southern Medical University Shenzhen Guangdong China; ^8^ Department of Pathology Affiliated Tumour Hospital of Xinjiang Medical University Urumqi Xinjiang China; ^9^ Department of Pathology Shenzhen Hospital The University of Hong Kong Shenzhen Guangdong China; ^10^ School of Pharmaceutical Sciences Guangdong Provincial Key Laboratory of New Drug Screening Southern Medical University Guangzhou Guangdong China; ^11^ School of Medicine Southern University of Science and Technology Shenzhen Guangdong China; ^12^ Guangdong Provincial Key Laboratory of Cell Microenvironment and Disease Research Shenzhen Guangdong China

**Keywords:** cervical cancer, N4‐acetylcytidine, NAT10/ac4C/FOXP1 axis, glycolysis, PD‐L1 blockade‐mediated immunosuppression

## Abstract

Immunotherapy has recently emerged as the predominant therapeutic approach for cervical cancer (CCa), driven by the groundbreaking clinical achievements of immune checkpoint inhibitors (ICIs), such as anti‐PD‐1/PD‐L1 antibodies. N4‐acetylcytidine (ac4C) modification, catalyzed by NAT10, is an important posttranscriptional modification of mRNA in cancers. However, its impact on immunological dysregulation and the tumor immunotherapy response in CCa remains enigmatic. Here, a significant increase in NAT10 expression in CCa tissues is initially observed that is clinically associated with poor prognosis. Subsequently, it is found that HOXC8 activated NAT10 by binding to its promoter, thereby stimulating ac4C modification of FOXP1 mRNA and enhancing its translation efficiency, eventually leading to induction of GLUT4 and KHK expression. Moreover, NAT10/ac4C/FOXP1 axis activity resulted in increased glycolysis and a continuous increase in lactic acid secretion by CCa cells. The lactic acid‐enriched tumor microenvironment (TME) further contributed to amplifying the immunosuppressive properties of tumor‐infiltrating regulatory T cells (Tregs). Impressively, NAT10 knockdown enhanced the efficacy of PD‐L1 blockade‐mediated tumor regression in vivo. Taken together, the findings revealed the oncogenic role of NAT10 in initiating crosstalk between cancer cell glycolysis and immunosuppression, which can be a target for synergistic PD‐1/PD‐L1 blockade immunotherapy in CCa.

## Graphical abstract

This study illustrates the oncogenic role of NAT10 in cervical cancer (CCa) and elucidates the mechanism by that the NAT10/ac4C/FOXP1 axis functions to accelerate glycolysis and promote infiltration of immunosuppressive Tregs into TME. Additionally, NAT10 knockdown contributes to enhancing PD‐L1 blockade efficacy as a combinatorial therapy for CCa, highlighting the potential of targeting NAT10‐mediated ac4C modification as a therapeutic strategy.

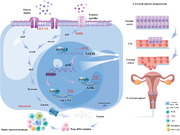

## 1 Introduction

Cervical cancer (CCa) ranks fourth in incidence and mortality worldwide and is the second most common malignancy among women in China.^[^ ^1^ ^]^ Emerging clinical evidence of early‐stage CCa has led to improvements in its prevention and diagnosis due to effective human papillomavirus (HPV) vaccination and screening strategies.^[^ ^2^ ^]^ However, despite treatment with the best available therapeutic regimens, patients with recurrent or metastatic (R/M) CCa have historically had a poor prognosis, with a 5‐year survival rate of only 17% over the past 30 decades, even after surgical resection, radiotherapy and chemotherapy.^[^ ^3^ ^]^ Recently, immunotherapy has been considered an efficient treatment option for R/M CCa.^[^ ^4^ ^]^ Studies of several immunotherapeutic approaches have verified that immune checkpoint blockade is a crucial clinical reality with remarkable outcomes.^[^ ^5^ ^]^ Indeed, treatment with ICIs targeting the PD‐1/PD‐L1 axis has become one of the most effective therapies for diverse cancers.^[^ ^6^ ^]^ Specifically, the PD‐1/PD‐L1 pathway attenuates T‐cell responses and promotes T‐cell tolerance in CCa.^[^ ^7^ ^]^ Thus, the TME, particularly the tumor immune microenvironment (TIME), exerts profound clinical impacts on the outcomes of immunotherapy in CCa. In 2018, the FDA approved pembrolizumab (Keytruda®, Merck) for the treatment of PD‐L1‐positive advanced CCa.^[^ ^8^, ^9^ ^]^ Nivolumab,^[^^10]^ targeting the receptor PD‐1, and atezolizumab,^[^ ^11^ ^]^ targeting the ligand PD‐L1 were also investigated in clinical trials (identifiers NCT02488759 and NCT02921269, respectively). Although PD‐L1 is expressed in 34.4%−96% of CCa tissues, the objective response rate was only 18% (95% CI, 11 to 28) among cemiplimab‐treated patients with PD‐L1 expression in ≥1% of cells and merely 11% (95% CI, 4 to 25) among those with PD‐L1 expression in <1% of cells.^[^ ^12^ ^]^ The results of these trials suggest that a small subset of PD‐L1‐positive patients benefit from PD‐1/PD‐L1 inhibitor therapy, underscoring the need for combinatorial approaches involving blockade of the PD‐L1 pathway for cancer therapy. Moreover, these observations emphasize the importance of developing strategies to modulate the TIME and identifying novel therapeutic targets to improve immunotherapy outcomes.

mRNAs can undergo ≈170 types of posttranscriptional modifications, which are catalyzed by dedicated and typically highly conserved enzymatic complexes. However, a variety of diseases result from the disruption of the enzymatic complex that catalyze these modifications, which include N6‐methyladenosine (m^6^A),^[^ ^13^ ^]^ N1‐methyladenosine (m^1^A),^[^ ^14^ ^]^ 5‐methylcytidine (m^5^C), N7‐methylguanosine (m^7^G) and N4‐acetylcytidine (ac4C).^[^ ^15^ ^]^ ac4C is often present on tRNAs and rRNAs as well as on human and yeast mRNAs, and it can increase translation efficiency through enrichment of C bases at the wobble position in codons and promote mRNA stability.^[^ ^16^ ^]^ Emerging evidence has suggested that ac4C modification may be associated with the development of several diseases, such as inflammatory diseases, metabolic diseases, autoimmune diseases, and diverse cancers.

As a member of the GCN5‐related NAT (GNAT) family of histone acetyltransferases, NAT10 serves as an enzymatic catalyst for ac4C modification on rRNAs, tRNAs, and mRNAs.^[^ ^16^ ^]^ Regarding its involvement in various cancers, NAT10 has been implicated in the pathogenesis of hepatocellular carcinoma,^[^ ^17^ ^]^ bladder cancer,^[^ ^18^ ^]^ and acute myeloid leukemia.^[^ ^19^ ^]^ Recently, Zhang et al. showed that NAT10 can regulate the ac4C level of COL5A1 mRNA through a direct interaction to accelerate epithelial–mesenchymal transition and metastasis of gastric cancer cells.^[^ ^20^ ^]^ Despite accumulating evidence demonstrating the effect of NAT10 on cancer development, the functional importance of the ac4C writer NAT10 in CCa remains an open area of investigation.

Dysregulation or alteration of energy metabolism is a fundamental hallmark of tumor cells, which depend on glycolysis for energy production.^[^ ^21^, ^22^ ^]^ The preference for aerobic glycolysis, also called the Warburg effect, is a quintessential characteristic of cancer cells.^[^ ^23^, ^24^ ^]^ Accumulating evidence has demonstrated that elevated lactate production is significantly correlated with recurrence and the metastatic potential of tumors, ultimately leading to a poor prognosis in patients.^[^ ^25^ ^]^ However, there are few reports regarding the underlying mechanisms and regulatory network of epigenetic factors, especially ac4C modification, involved in glycolytic metabolism during CCa tumorigenesis.

The intricate TME comprises not only tumor cells but also diverse types of immune cells, including CD4+ FOXP3+ Tregs, myeloid‐derived suppressor cells (MDSCs) and tumor‐associated macrophages (TAMs). Importantly, a recent study demonstrated that elevated levels of lactic acid can inhibit CD8+ T‐cell proliferation and reduce cytokine production.^[^ ^26^ ^]^ Tregs can express MCT1, a lactic acid transporter, to increase lactate uptake.^[^ ^27^ ^]^ Intriguingly, high lactic acid secretion increases PD‐1 expression and the suppressive activity of Tregs, leading to failure of PD‐1 blockade therapy; this observation suggests that lactate within the TME plays an indispensable role in modulating the function of Tregs through upregulation of PD‐1 expression.^[^ ^27^ ^]^

Here, we aimed to investigate the role of NAT10‐driven metabolic and immune reprogramming in CCa and elucidate the mechanism by which the NAT10/ac4C/FOXP1 axis in CCa functions to accelerate glycolysis and lactate production, which ultimately promotes the infiltration of immunosuppressive Tregs into the TME. Additionally, we first showed that NAT10 knockdown contributed to enhancing PD‐L1 blockade efficacy as a combinatorial therapy for CCa, highlighting the potential of targeting NAT10‐mediated ac4C modification as a promising immunotherapeutic strategy.

## 2 Results

### 2.1 NAT10 Plays a Potential Oncogenic Role in CCa

**Figure 1:**
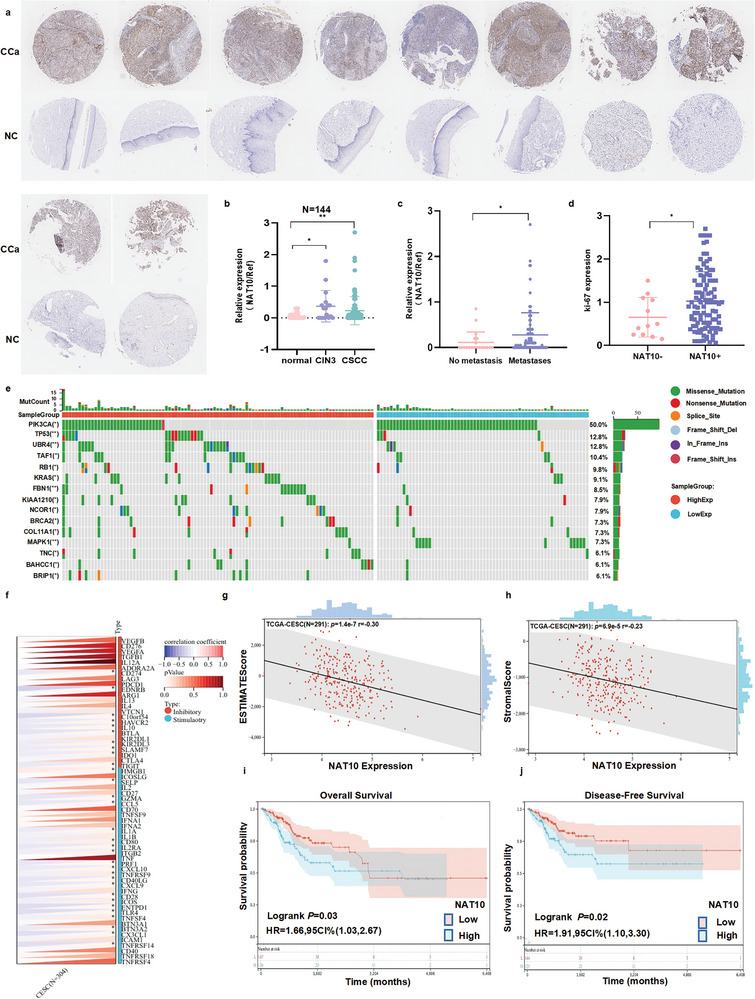
NAT10 plays a potential oncogenic role in CCa. a) Representative immunohistochemical images of NAT10 expression in CCa (ten cases) and normal cervical tissue (ten cases) in the TMA. (b) TMA analysis (n = 144) showed the upregulation of NAT10 in CCa tissues, as shown in the histogram. The lines indicate the mean and SD (one‐way ANOVA). c,d) Analysis of clinical data of patients showed that NAT10 expression was positively correlated with lymph node metastasis and the expression of Ki‐67 in CCa tissues. e) The mutational landscape of selected key genes, including TP53 and KRAS, upon NAT10 upregulation in CCa cells was analyzed by SangerBox. f) Correlation analysis between NAT10 expression and key immune checkpoint pathways in CCa tissues and g,h) ESTIMATE analysis through SangerBox showed that elevated NAT10 expression was associated with a lower EstimateScore (g) and StromalScore (h) in CCa. i,j) Kaplan‒Meier analysis showed that higher expression of NAT10 was associated with shorter OS (i) and DFS (j) times in CCa patients; p = 0.03 and p = 0.02 by the log‐rank test, respectively. The data are shown as the means ± SDs; *p<0.05; **p<0.01; *** *p*<0.001; **** *p*<0.0001.

First, we verified the in silico findings by analyzing a TMA consisting of 17 samples from patients with cervical intraepithelial neoplasia(CIN), 95 samples from patients with CCa and 32 samples from control individuals based on the pathological diagnosis. Importantly, our results confirmed the upregulation of NAT10 protein expression in CCa specimens (**Figure** **1**a), and this aberrant upregulation was associated with less favorable clinicopathological characteristics, such as high tumor stage (*P*<0.05) (Figure 1b) and lymph node metastasis (*P*<0.05), in CCa (Figure 1c). Moreover, increased NAT10 expression was positively correlated with increased Ki‐67 expression (*P*<0.05) (Figure 1d), which is a marker for cancer cell proliferation and poor prognosis.

Then, The Cancer Genome Atlas (TCGA) pancancer database was analyzed using SangerBox (http://vip.sangerbox.com/home.html), revealing frequent instances of high NAT10 expression in various cancers (Figure S1a, Supporting Information) and higher levels of NAT10 expression in CCa tissues (n = 304) than in normal cervical epithelial tissues (n = 13). Interestingly, to describe the mutational landscape of CCa patients with high NAT10 expression, the mutational landscape (insertions/deletions/single‐nucleotide variants) was compared between the NAT10‐high and NAT10‐low groups stratified based on the median level of NAT10 expression. For this analysis, the chi‐square test was used to analyze data from patients with CCa downloaded from the TCGA database through SangerBox. The results showed significantly higher mutation rates of key genes such as TP53, UBR4, KRAS and FBN1 in CCa tissues with upregulated NAT10 expression than in CCa tissues with low expression of NAT10 (Figure 1e). Obviously, NAT10 expression in CCa was closely related to key immune checkpoint pathways. ESTIMATE analysis further demonstrated that NAT10 upregulation was associated with a lower ESTIMATE score and stromal score in CCa, indicating the pivotal role of NAT10 in regulating tumor immunity in CCa (Figure 1f–h). To investigate the impact of NAT10 on disease‐free survival (DFS) and overall survival (OS), we then retrieved the survival data of CCa patients from the TCGA database. Kaplan–Meier survival analysis revealed that high expression of NAT10 was weakly correlated with OS in CCa patient (*P* = 0.03) and was linked to shortened DFS (*P* = 0.02) (Figure 1i,j) [log‐rank (Mantel–Cox) P ¼ 0.0075]. These results collectively suggest that overexpression of NAT10 is a prognostic factor and has potential oncogenic implications in CCa tumorigenesis (Table S1, Supporting Information).

### 2.2 The Transcription Factor (TF) HOXC8 Activates NAT10 Transcription in CCa

**Figure 2:**
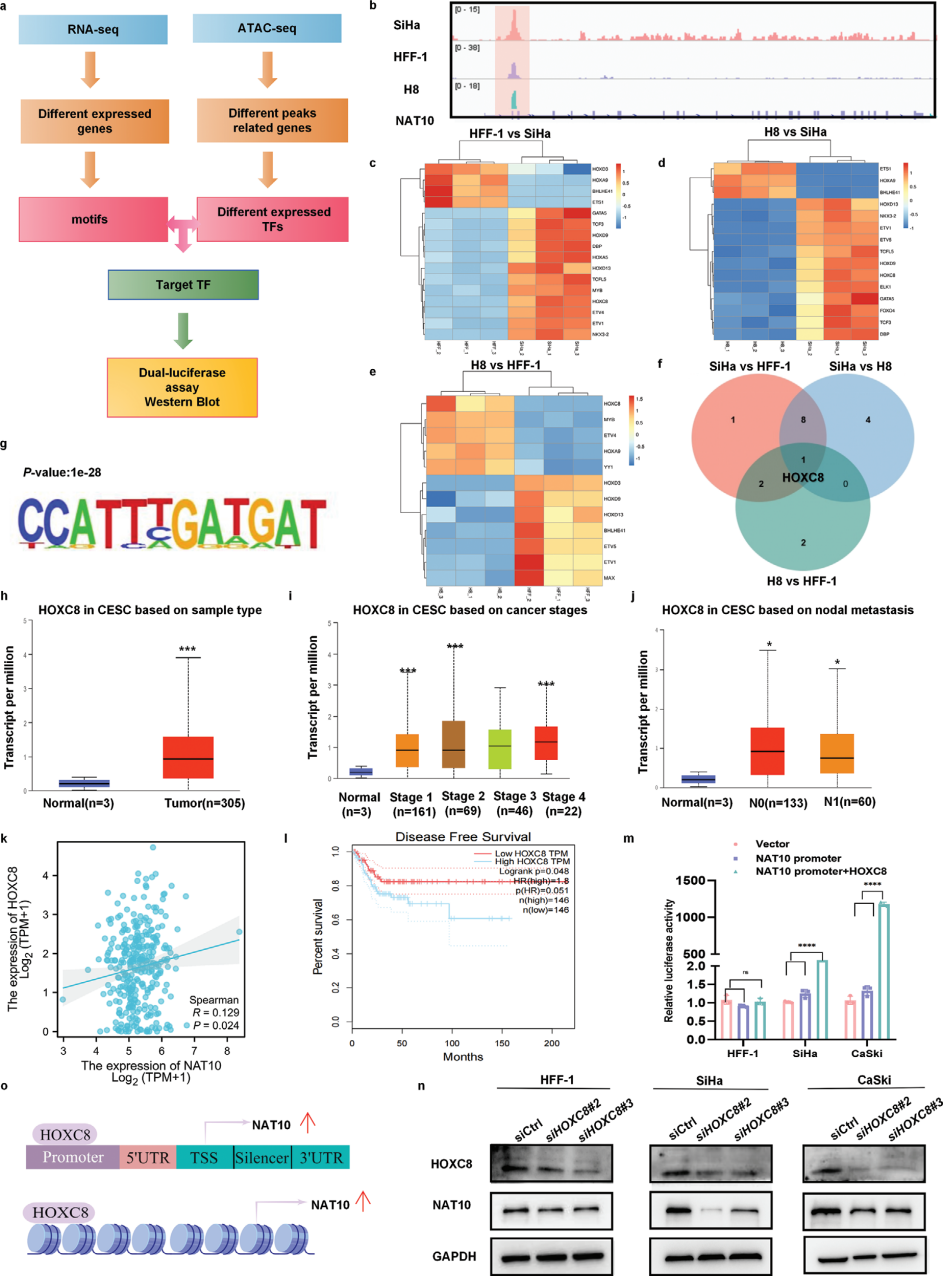
TheTF HOXC8 activates NAT10 transcription in CCa. a) HOXC8 screening strategy and HOXC8 analyses in CCa. b) IGV plot of ATAC‐seq stacked reads at NAT10 in HFF‐1, H8, and SiHa cells. c–e) Integrated analysis of ATAC‐seq and RNA‐seq data, with a heatmap displaying differentially expressed TFs that may modulate NAT10 transcription in the SiHa versus HFF‐1, SiHa versus H8 and H8 versus HFF‐1 groups. f) Venn diagram showing the unique TFs with a consistent trend in the four comparisons described above. g) The motif detected by MEME motif analysis of the ATAC‐seq data, showing the potential HOXC8 binding sites in the NAT10 promoter. h–j) Analysis of TCGA data through UALCAN showed that HOXC8 mRNA expression was significantly upregulated (h) (n = 308) and positively associated with characteristics related to tumor development, including tumor stage i) (n = 301) and nodal metastasis j) (n = 196), in CCa tissues. k) The correlation of NAT10 and HOXC8 expression was analyzed through the TCGA database using the tool at the following site: https://www.xiantao.love/. l) Kaplan‐Meier survival curve demonstrating that HOXC8 upregulation in CCa was associated with shorter DFS times. m) Dual‐luciferase assay results showing a significant increase in the firefly luciferase/Renilla luciferase ratio in CCa cells transfected with the NAT10 promoter and HOXC8 plasmids compared with that in cells transfected with only the NAT10 promoter or with the vector. n) HOXC8 silencing resulted in downregulation of NAT10 in SiHa and CaSki cells compared with HFF‐1 cells, based on Western blot analysis. o) Image generated with Figdraw showing that HOXC8 functions as a TF to upregulate NAT10 in CCa cells. The data are shown as the means ± SDs; *p<0.05; **p<0.01; *** *p*<0.001; **** *p*<0.0001.

ATAC‐seq can be used to generate comprehensive maps of chromatin accessibility and is increasingly recognized as a powerful tool for understanding systematic, global features; the dynamics of gene expression regulation; and the complexities of TF binding.^[^ ^28^ ^]^ Integrated analysis of RNA‐seq and ATAC‐seq data can reveal factors related to motif protection and determine the relationship between the accessibility of a regulatory element and the expression of the predicted target gene(s).^[^ ^29^ ^]^ To elucidate the mechanism underlying NAT10 upregulation in CCa, we identified the relevant TFs by integrating ATAC‐seq footprinting and RNA‐seq data (**Figure** **2**a). Because more than 90% of CCa cases are due to infection with high‐risk HPV types, we utilized Integrated Genomics Viewer (IGV) to identify signals indicating accessible chromatin surrounding the transcription start site (TSS) of *NAT10* and aimed to define TF candidates at regions of accessible chromatin in HFF‐1 (noncancerous control), H8 (noncancerous with HPV16 E6 and E7) and SiHa (CCa cells with HPV16 E6 and E7) cells (Figure 2b). By utilizing the HOMER algorithm for de novo motif discovery, we identified 32 TF motifs and narrowed our focus to those that were enriched in SiHa cells. The RNA‐seq heatmap generated from the three different groups displayed several potential TFs that may regulate NAT10 expression (Figure 2c–e). Through overlapping the differentially expressed TFs across the four comparison groups, HOXC8 emerged as a uniquely overrepresented TF in SiHa cells (Figure 2f,g). Furthermore, TCGA analysis showed significant upregulation of HOXC8 mRNA expression in CCa (*P*<0.05) (Figure 2h), which was positively associated with cancer stage and lymph node metastasis (*P*<0.05) (Figure 2i,j). Notably, Gene Expression Profiling Interactive Analysis (GEPIA) demonstrated that the mRNA level of HOXC8 was positively correlated with the NAT10 expression level in CCa (*P*<0.05) (Figure 2k), while high levels of HOXC8 mRNA expression were also significantly associated with short DFS times (*P*<0.05) (Figure 2l).^[^ ^30^ ^]^

To clarify the effects of this key TF on *NAT10* transcription, a dual‐luciferase assay was performed. Cotransfection with the HOXC8 and NAT10 promoter plasmids significantly increased luciferase activity, as evidenced by the firefly luciferase/Renilla luciferase ratios, which were decreased in both CCa cell lines after transfection with either the vehicle control plasmid or the NAT10 promoter plasmid alone (Figure 2m). Consistent with these findings, the Western blot results demonstrated conspicuous suppression of NAT10 expression in CCa cells upon introduction of a specific siRNA targeting HOXC8 (Figure 2n). Collectively, these data strongly suggest that HOXC8 potentially increases NAT10 expression in CCa cells by binding to its promoter region and promoting its transcription (Figure 2o).

### 2.3 NAT10 Knockdown Inhibits CCa Cell Proliferation, Invasion, and Metastasis In Vitro and In Vivo

**Figure 3:**
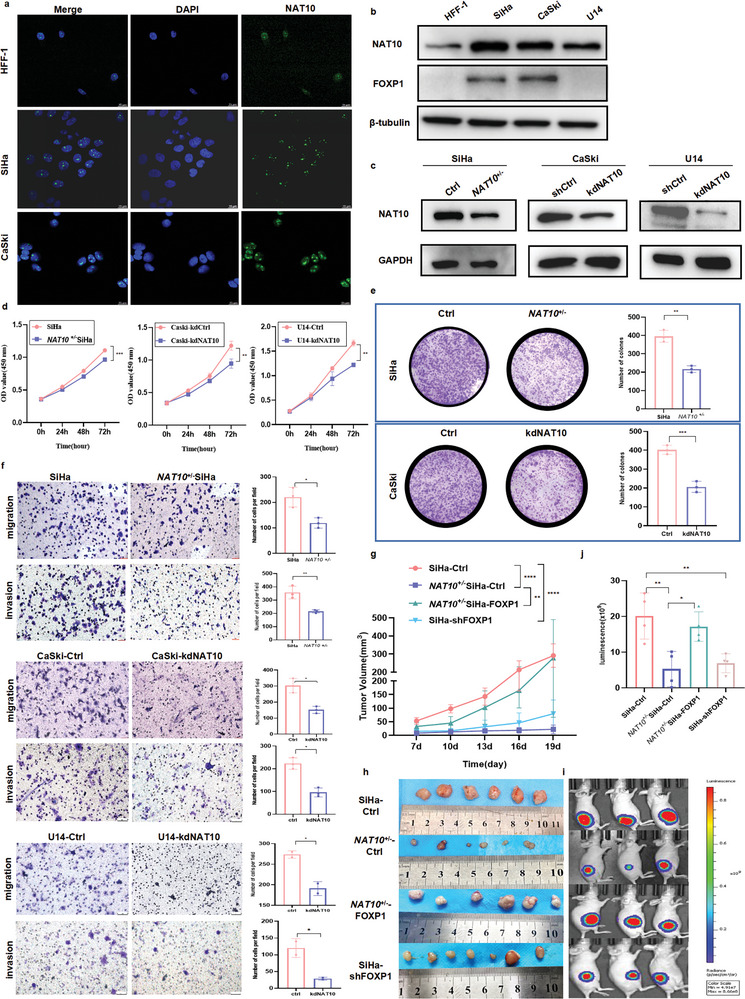
NAT10 knockdown inhibits CCa cell proliferation, invasion and metastasis in vitro and in vivo. a) Immunofluorescence staining showed the localization of NAT10 in CCa and HFF‐1 cells. b,c) Western blot analysis revealed the expression of NAT10 and FOXP1 in different cell lines (b) and verified the successful knockdown of NAT10 in CCa cells (c). d–f) NAT10 knockdown impaired CCa cell proliferation, as shown by the CCK8 assay (d), and impaired the colony formation (e) and the migration and invasion abilities of CCa cells, as shown by Transwell assays (f). g,h) Growth curves showing CCa cell proliferation in vivo (g) and the volume of tumors in different groups on the 21st day after cell inoculation (h) (n = 6). i,j) Representative fluorescence image i) and quantification of subcutaneous tumors fluorescence intensity (j) in each group on the 21st day after cell inoculation. The data are shown as the means ± SDs; *p<0.05; **p<0.01; *** *p*<0.001; **** *p*<0.0001; ns, nonsignificant.

As shown in **Figure** **3**a,b, the NAT10 level in CCa cells was significantly higher than that in HFF‐1 cells. Immunofluorescence assays further showed that NAT10 was localized predominantly in the nucleus and did not undergo nuclear export. To elucidate the functional role of NAT10 in CCa, we successfully established a cell line with stable NAT10 heterozygous knockdown (*NAT10* ^+/‐^ SiHa) with the CRISPR/Cas9 gene editing system and employed sgRNAs to knock down NAT10 expression in both CaSki and U14 CCa cells (Figure 3c). However, remodelin efficiently inhibited NAT10 activity in both CaSki and SiHa cells without affecting its expression, as remodelin is a specific inhibitor of NAT10 (Figure S1b). Depletion or inhibition of NAT10 significantly suppressed cell proliferation, as shown by the CCK‐8 assay (Figure 3d; Figure S1c, Supporting Information), while the colony formation assay revealed that the clonogenic capacity was greatly weakened by NAT10 knockdown in vitro (Figure 3e; Figure S1d, Supporting Information). Furthermore, NAT10 knockdown resulted in significant induction of G1 arrest (Figure S1e, Supporting Information). Moreover, inhibition or knockdown of NAT10 effectively suppressed the migration and invasion of CCa cells, as demonstrated by the Transwell assay (Figure 3f; Figure S1f, Supporting Information).

To further ascertain whether NAT10‐induced proliferation of CCa cells in vitro endows tumor growth ability in vivo, we established a subcutaneous xenograft model in nude mice using SiHa cells with stable silencing of NAT10 (*NAT10* ^+/‐^ SiHa) tagged with firefly luciferase (‐luc). Downregulation of NAT10 markedly impaired tumor growth in mice injected subcutaneously with either SiHa or *NAT10* ^+/‐^ SiHa cells (Figure 3g). At the end of the in vivo study, the tumors were excised, and a decreased growth rate of *NAT10* knockdown CCa tumors was observed (Figure 3h). Furthermore, continuous tumor growth was evidenced by the significant weekly increase in the luciferase signal intensity, which was detectable through live animal imaging (Figure 3i,j). These observations confirm that NAT10 exerts an oncogenic effect and could facilitate CCa cell proliferation, invasion and metastasis.

### 2.4 FOXP1 is the Target of NAT10 for ac4C Modification, and its Translation Efficiency is Enhanced by NAT10 in an ac4C‐Dependent Manner

**Figure 4:**
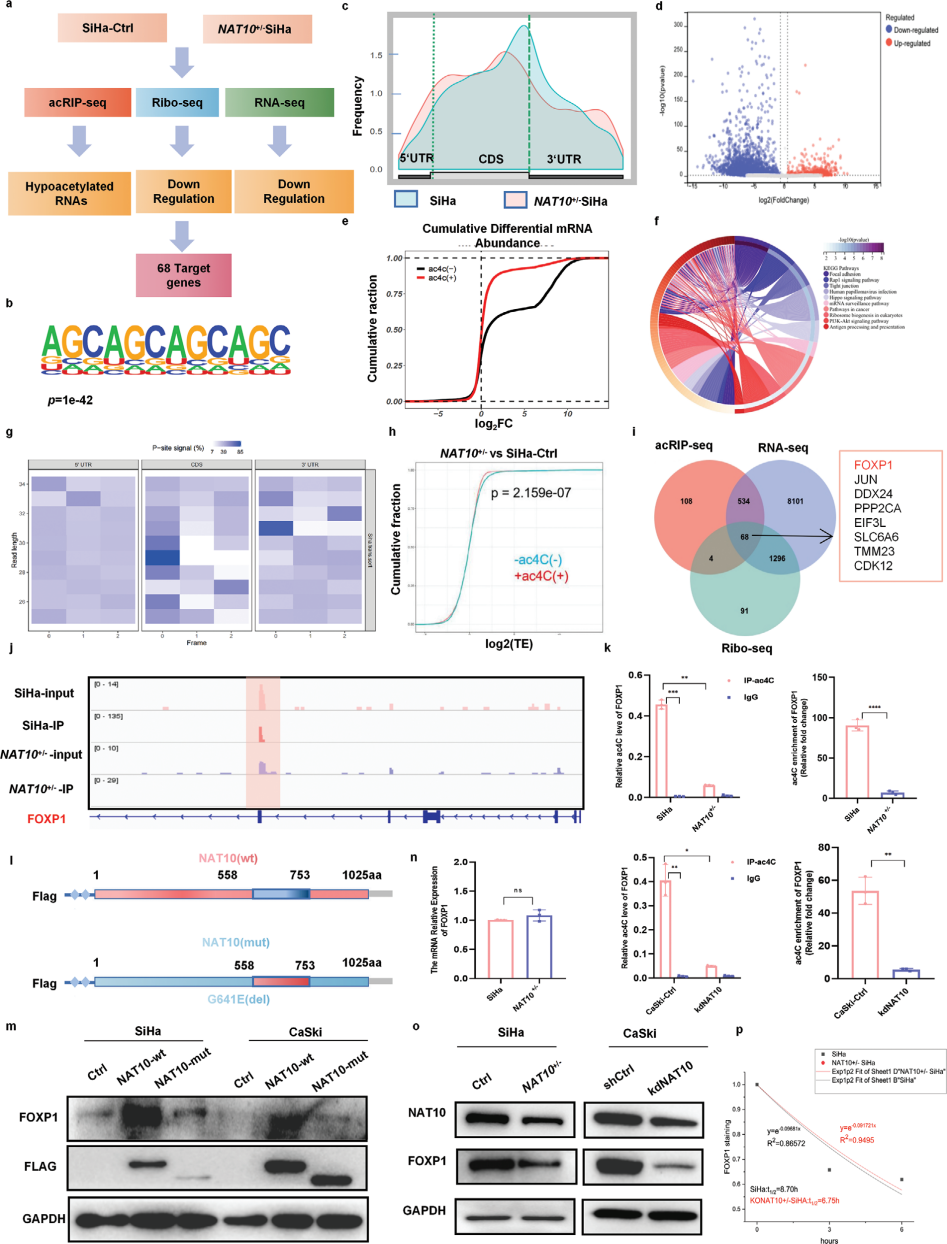
FOXP1 is the target of NAT10 for ac4C modification, and its translation efficiency is enhanced by NAT10 in an ac4C‐dependent manner. a) Flow chart of the screening and validation of the correlation between the expression of NAT10 and that of its downstream target FOXP1. b) The ac4C motif detected by MEME motif analysis of acRIP‐seq data. c) The distribution of acetylated peaks on mRNA. d) Volcano plot showing the change in the ac4C level on mRNA after NAT10 knockdown in SiHa cells. e) Cumulative distribution function plot showing differential expression of ac4C(–) and ac4C(+) transcripts in *NAT10* ^+/−^SiHa versus Ctrl cells. f) KEGG analysis results showing the pathway enrichment of genes with different ac4C levels. g) Heatmap showing the reading frame (RF) distribution in the annotated gene region. h) CDF plots of mRNA‐normalized ribosome footprint reads for ac4C(+) transcripts and ac4C(‐) transcripts in *NAT10* ^+/−^ SiHa versus control cells. i) Venn diagram showing the target genes with changes in ac4C acetylation, modification, transcription and translation levels after NAT10 knockdown. j) IGV software was used to visualize the peaks with ac4C enrichment in Ctrl and *NAT10* ^+/‐^ SiHa cells. k) The acRIP‐qPCR assay revealed the ac4C abundance on FOXP1 mRNA in Ctrl and *NAT10* ^+/−^ SiHa cells. l) Schematic showing the mutation site (del 558–753aa) in the NAT10‐mut vector and the NAT10‐wt vector. m) Western blot analysis was performed to measure the expression of Flag‐NAT10 and FOXP1 in CCa cells transfected with control, Flag‐NAT10‐wt or Flag‐NAT10‐mut plasmids for 48 h. n,o) mRNA (n) and protein (o) levels of FOXP1 in Ctrl and *NAT10* ^+/‐^ SiHa cells. p) The half‐life (t1/2) of FOXP1 mRNA remained unchanged after treatment with Act D for 0, 3, or 6 h in *NAT10* ^+/‐^ and SiHa cells. The data are shown as the means ± SDs; *p<0.05; **p<0.01; *** *p*<0.001; **** *p*<0.0001; ns, nonsignificant.

According to previous reports, NAT10 has been identified as the “writer” of ac4C modification in eukaryotes.^[^ ^15^ ^]^ However, the exact function of the ac4C modification mediated by NAT10 in CCa remains to be elucidated. Herein, we performed acRIP‐seq, RNA‐seq and Ribo‐seq on wild‐type and *NAT10* ^+/‐^ SiHa cells to identify ac4C‐modified transcripts in CCa cells and reveal the molecular mechanism by which ac4C modification promotes CCa progression (**Figure** **4**a).

Through acRIP‐seq analysis, we identified 808 ac4C peaks and discovered by utilizing the MEME algorithm that the CXXCXXCXX motif was highly enriched in SiHa cells (Figure 4b). Obviously, these ac4C peaks were predominantly located near protein‐coding transcripts and particularly enriched near start and stop codons (≈25% of peaks). Moreover, the ac4C distribution decreased from 34.9% to 31.9% in coding regions and increased from 37.4% to 45.5% in the 3′UTR in cells with low NAT10 expression compared to SiHa cells (Figure 4c). Furthermore, annotation and analysis of the ac4C peaks significantly altered by NAT10 knockdown revealed a significant decrease in the overall ac4C level of mRNA in SiHa cells with NAT10 silencing (Figure 4d,e). Moreover, Kyoto Encyclopedia of Genes and Genomes (KEGG) enrichment analysis was performed to analyze the differentially expressed genes (DEGs), which exhibited significant enrichment in the following pathways: cell adhesion, pathways in cancer, PI3K‐Akt signaling pathway, mRNA surveillance pathway, and HPV infection pathway (Figure 4f). This finding suggests that ac4C modification of RNA may be involved in the regulation of CCa development.

Furthermore, we used Ribo‐seq to assess the ribosome loading of each mRNA as represented by ribosome‐protected reads (Figure 4g). The results of Ribo‐seq analysis indicated that a reduced level of ac4C modification led to transcriptional downregulation due to a decreased translation efficiency (Figure 4h). Intriguingly, among the 68 mRNAs, FOXP1 mRNA exhibited the most obvious alteration upon NAT10 downregulation, with reductions of 6.39‐, 5.12‐, and 2.78‐fold in the transcript abundance determined by acRIP‐seq, Ribo‐seq and RNA‐seq (Figure 4i). Therefore, considering the role of ac4C modification in CCa and the identification of ac4C‐modified peaks on RNA through visualization with IGV software (Figure 4j), we speculated that FOXP1 may be a key target of NAT10 in CCa cells.

To validate whether FOXP1 mRNA is a direct target of ac4C modification in CCa cells, acRIP‐qPCR was conducted. Marked enrichment of ac4C on FOXP1 mRNA was observed, and the ac4C level on FOXP1 mRNA was significantly reduced (approximately 10‐fold) in NAT10‐depleted CCa cells compared to control cells (Figure 4k). Additionally, we constructed plasmids containing the sequence of wild‐type FLAG‐tagged NAT10 (NAT10‐wt) or NAT10 with a mutated N‐acetyltransferase modification site (del 558–753aa) (NAT10‐mut) (Figure 4l). Introduction of NAT10‐wt but not NAT10‐mut increased FOXP1 expression (Figure 4m). Thus, collectively, these results indicate that NAT10‐mediated ac4C modification plays a vital role in the upregulation of FOXP1 in CCa cells.

However, NAT10 silencing resulted in a significant decrease in the FOXP1 protein level without affecting its mRNA level in CCa cells (Figure 4n,o). Thus, we assessed the stability of individual mRNAs in CCa cells and found that NAT10 knockdown did not significantly affect FOXP1 transcript stability (Figure 4p). Considering the Ribo‐seq results, these data collectively suggest that NAT10‐mediated ac4C modification may regulate the translational efficiency of FOXP1 rather than its mRNA stability.

### 2.5 FOXP1 Overexpression Reverses the Effect of NAT10 Deficiency on the Malignant Phenotype of CCa Cells

**Figure 5:**
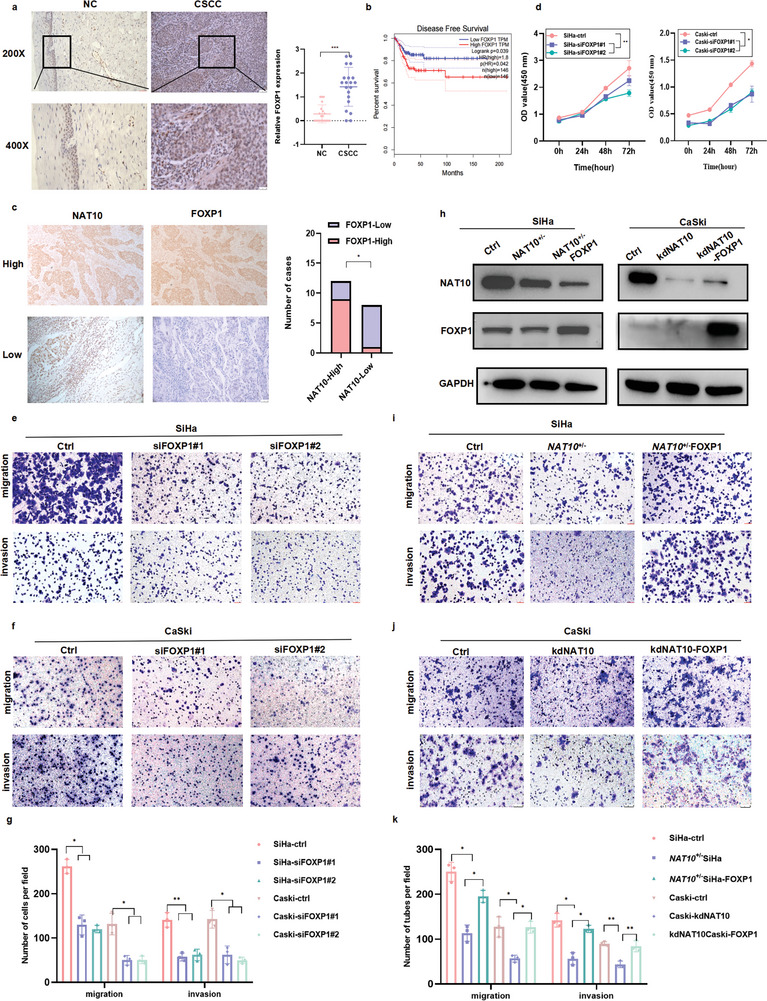
FOXP1 overexpression reverses the effect of NAT10 deficiency on the malignant phenotype of CCa cells. a,c) Immunohistochemical results demonstrating the upregulation of FOXP1 in CCa (n = 20) versus NC tissues (n = 20) (a) and the positive correlation between FOXP1 and NAT10 expression in CCa patients (n = 20) (c). b) Elevated FOXP1 expression was associated with short DFS in CCa patients, based on the results of Kaplan‒Meier analysis in GEPIA. d–g) The role of FOXP1 in CCa was explored through a CCK8 assay (d) and Transwell migration and invasion assays (e‐g). h) Western blot analysis was performed to measure the protein levels of NAT10 and FOXP1 in NAT10‐deficient SiHa and CaSki cells upon overexpression of FOXP1. i–k) Transwell assay results showing that FOXP1 overexpression restored the migration and invasion abilities of NAT10 knockdown CCa cells. The data are shown as the means ± SDs; *p<0.05; **p<0.01; *** *p*<0.001; **** *p*<0.0001; ns, nonsignificant.

Since the functions of FOXP1 in CCa remain unclear, we initially compared the protein level of FOXP1 in 20 CCa tissues with that in 20 normal cervical epithelial tissues and found that the FOXP1 protein level was significantly increased in in CCa tissues compared to their normal counterparts (*P<*0.01) (**Figure** **5**a). Kaplan‐Meier analysis through GEPIA showed that CCa patients with increased FOXP1 mRNA expression exhibited a worse DFS prognosis (*P* = 0.039) (Figure 5b). Most importantly, the FOXP1 expression level exhibited a positive correlation with the level of NAT10 in CCa tissues (Figure 5c). In addition, FOXP1 was overexpressed in SiHa and CaSki cells compared with HFF‐1 cells, while FOXP1 expression was low in U14 cells (Figure 3b). To further characterize the oncogenic function of FOXP1 in CCa, we designed and used two different siRNAs to target FOXP1 in SiHa and CaSki cells, and we also generated FOXP1‐overexpressing U14 cells (oeFOXP1‐U14) (Figure S2a, Supporting Information). Knockdown of FOXP1 dramatically suppressed CCa cell proliferation (Figure 5d), migration and invasion (Figure 5e–g). However, there was no significant difference in cell viability between U14‐Ctrl and U14‐oeFOXP1 cells, indicating that the differential expression of FOXP1 in humans and mice may lead to distinct phenotypes (Figure S2b, Supporting Information).

To further validate the role of FOXP1 in the NAT10‐mediated promotion of CCa progression, we conducted overexpression experiments on SiHa and CaSki cells with NAT10 knockdown (Figure 5h; Figure S2c, Supporting Information). Importantly, the results of Transwell migration and Matrigel invasion assays showed that FOXP1 overexpression effectively restored the migration and invasion abilities of NAT10 knockdown CCa cells (Figure 5i–k). Moreover, a subcutaneous tumorigenesis model was established using shFOXP1 and *NAT10* ^+/‐^ FOXP1 SiHa cells. Consistent with the in vivo results, the results in this model confirmed that overexpression of FOXP1 can attenuate the suppressive effect of NAT10 knockdown on tumor growth in vivo (Figure 3g–j), further emphasizing the crucial role of ac4C modification catalyzed by NAT10 in CCa tumor development. Collectively, these data underscore the oncogenic function of upregulated FOXP1 in CCa malignant progression; thus, FOXP1 upregulation could ameliorate the tumor‐suppressive effect of NAT10 depletion in CCa cells.

### 2.6 The NAT10/ac4C/FOXP1 Axis Enhances Glycolysis by Targeting GLUT4 and KHK

**Figure 6:**
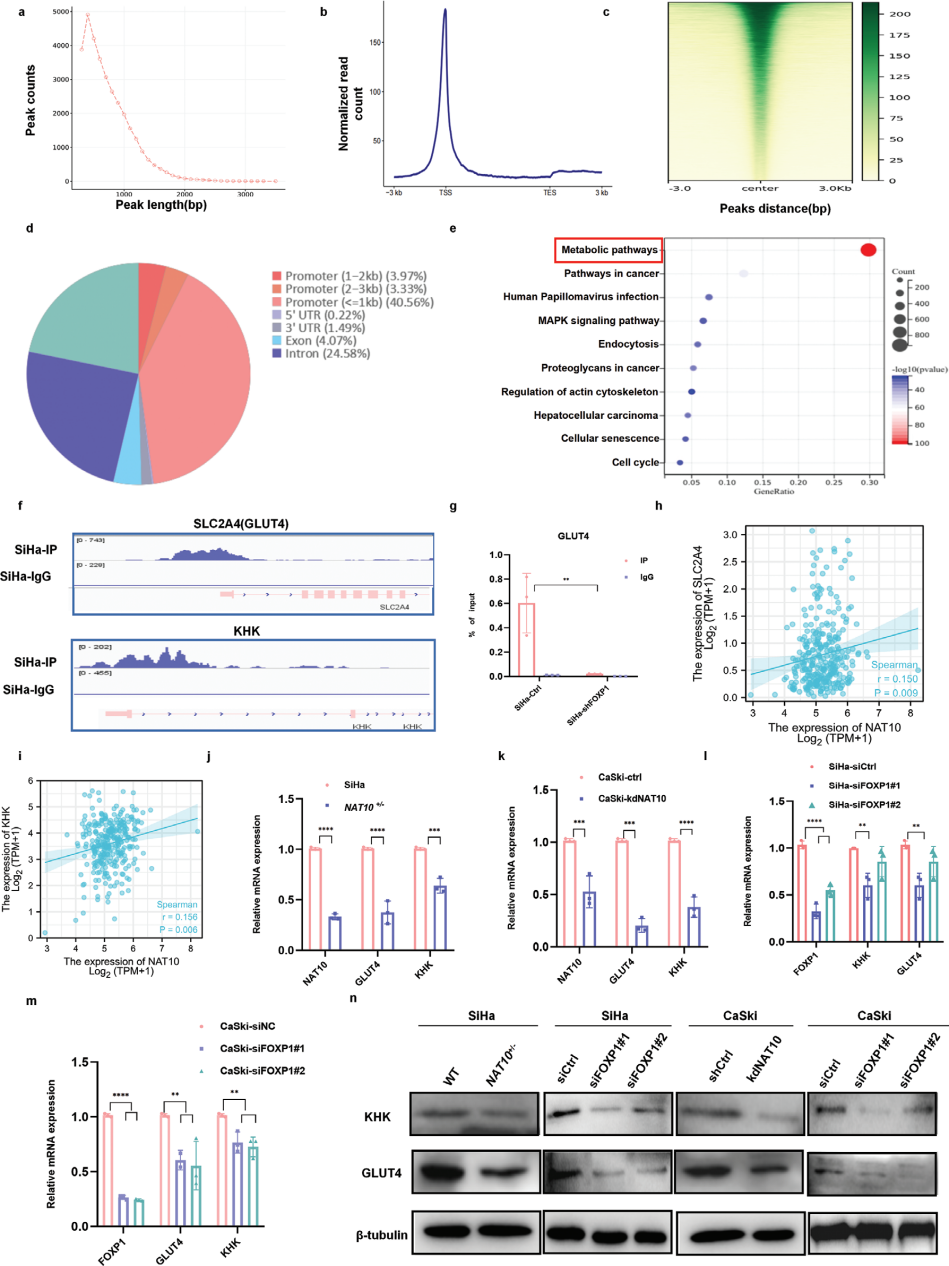
The NAT10/ac4C/FOXP1 axis enhances glycolysis by targeting GLUT4 and KHK. a) Distribution diagram showing the size of peaks. b) Reads were mainly enriched near the TSS. c) Heatmap of the central peak signals indicating that the signals of the enriched sites were concentrated in the TSS region. d) Pie chart showing the distribution of peaks in the functional regions of the genes. e) KEGG analysis results showing the pathways in which target genes may be enriched. f,g) IGV software visualization (f) and ChIP‒qPCR results (g) showing the FOXP1‐enriched peaks in GLUT4 and KHK in SiHa cells. h,i) The correlations between NAT10 expression and GLUT4 (SLC2A4) and KHK expression were evaluated using tools at https://www.xiantao.love/. j–n) Knockdown of NAT10 or FOXP1 reduced GLUT4 and KHK expression in SiHa and CaSki cells, as determined by qRT‒PCR and Western blot analyses. The data are shown as the means ± SDs. *p<0.05; **p<0.01; *** *p*<0.001; **** *p*<0.0001; ns, nonsignificant.

Considering the ability of FOXP1 to localize to the nucleus and act as a transcriptional regulator,^[^ ^31^ ^]^ we speculated that FOXP1 may be able to bind these related gene promoter regions and transcriptionally activate their expression. Utilizing CUT&Tag, which generates efficient high‐resolution sequencing libraries for profiling diverse chromatin components, we determined that nearly all peaks observed in SiHa cells covered less than 1000 bp (**Figure** **6**a) and showed moderately strong signals across the entire set of sites, particularly at TSSs (Figure 6b). Furthermore, the signal values of all peaks were meticulously calculated, and a heatmap was generated to demonstrate that the signals were strongly concentrated near enriched sites (Figure 6c), with the most enrichment within the gene promoter region (promoter ≤ 1000 bp) (Figure 6d). Through identification of cellular metabolic processes of the FOXP1‐binding genes via GO and KEGG analyses (Figure 6e), we postulated that FOXP1 may play a pivotal regulatory role in glucose metabolism, prompting us to investigate key enzymes involved in glycolytic metabolism.

Interestingly, CUT&Tag sequencing data indicated robust enrichment of FOXP1 in the promoters of GLUT4 and KHK (Figure 6f), which participate in glycolysis, as extensively documented in many publications.^[^ ^32^, ^33^ ^]^ The ChIP‒qPCR results further demonstrated greatly decreased enrichment of FOXP1 signals in the promoter of GLUT4 upon knockdown of FOXP1 (Figure 6g). Furthermore, the expression of GLUT4 was positively correlated with that of FOXP1 and NAT10 and the expression of KHK was positively correlated with that of NAT10 in CCa, based on analysis of TCGA datasets through GEPIA (Figure 6h,i; Figure S2d, Supporting Information). Furthermore, depletion of either NAT10 or FOXP1 resulted in suppression of GLUT4 and KHK expression at both the transcriptional and translational levels, as confirmed by qRT‒PCR (Figure 6j–m) and Western blotting (Figure 6n), suggesting that FOXP1 modulates the expression of GLUT4 and KHK.

**Figure 7:**
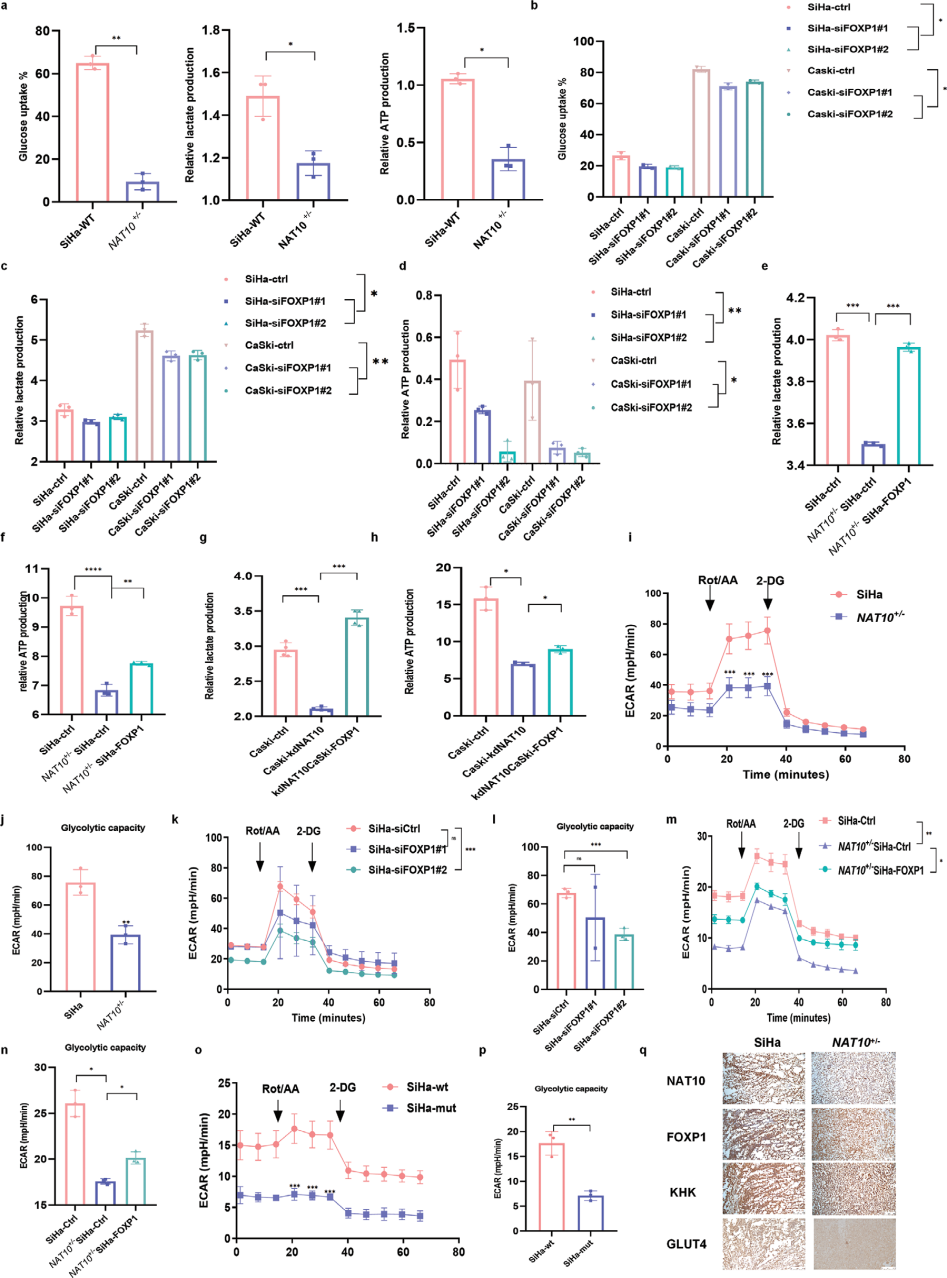
NAT10/ac4C/FOXP1 axis activity induced an increase in glycolysis in CCa. a–d) The decreased glucose uptake and lactate and ATP production in NAT10‐knockdown SiHa (a) and FOXP1‐silenced CCa cells b–d) compared with control cells. e–h) Increased Lactate and ATP production in NAT10‐deficient SiHa and CaSki cells upon overexpression of FOXP1. i–p) ECAR measurement was performed in the indicated groups of cells, as demonstrated above, after injection of metabolic inhibitors at different time points. q) Immunohistochemical staining showed the expression of NAT10, FOXP1, GLUT4, and KHK in SiHa and *NAT10* ^+/−^ subcutaneous tumors. The data are shown as the means ± SDs. *p<0.05; **p<0.01; *** *p*<0.001; **** *p*<0.0001; ns, nonsignificant.

To investigate the role of NAT10 in modulating glycolysis to promote malignant behaviors in CCa cells, glycolytic flux was evaluated by measuring the extracellular acidification rate (ECAR), glucose consumption, lactate production, and ATP level. As shown in **Figure** **7**a; Figure S2e (Supporting Information), NAT10‐depleted CCa cells exhibited significantly reduced glucose consumption as well as decreased lactate and ATP production. Consistent with these findings, FOXP1 silencing also led to decreases in glucose consumption and lactate and ATP production (Figure 7b–d; Figure S2f, Supporting Information), whereas the glycolytic ability was restored when FOXP1 was overexpressed in the NAT10 knockdown background (Figure 7e–h). Furthermore, the results of ECAR measurement not only validated the decrease in glycolysis in CCa cells with NAT10 or FOXP1 knockdown (Figure 7i–l; Figure S2g–i, Supporting Information) but also showed that overexpression of FOXP1 reversed this effect (Figure 7m,n; Figure S2j, Supporting Information). However, a consistently lower level of glycolytic metabolism was observed in CCa cells transfected with NAT10‐mut (Figure 7o,p), indicating that the ac4C modification catalyzed by NAT10 has a crucial effect on promoting glycolysis in CCa. Moreover, SiHa tumor xenografts established from cells with NAT10 deficiency showed significantly decreased staining for NAT10, FOXP1, GLUT4, and KHK (Figure 7q). In summary, our findings reveal that upregulation of FOXP1 induced by ac4C modification enhances glycolytic activity by activating GLUT4 and KHK transcription in CCa cells.

### 2.7 NAT10/ac4C/FOXP1 Axis Activity Increases Treg Infiltration in Highly Glycolytic Tumors

**Figure 8:**
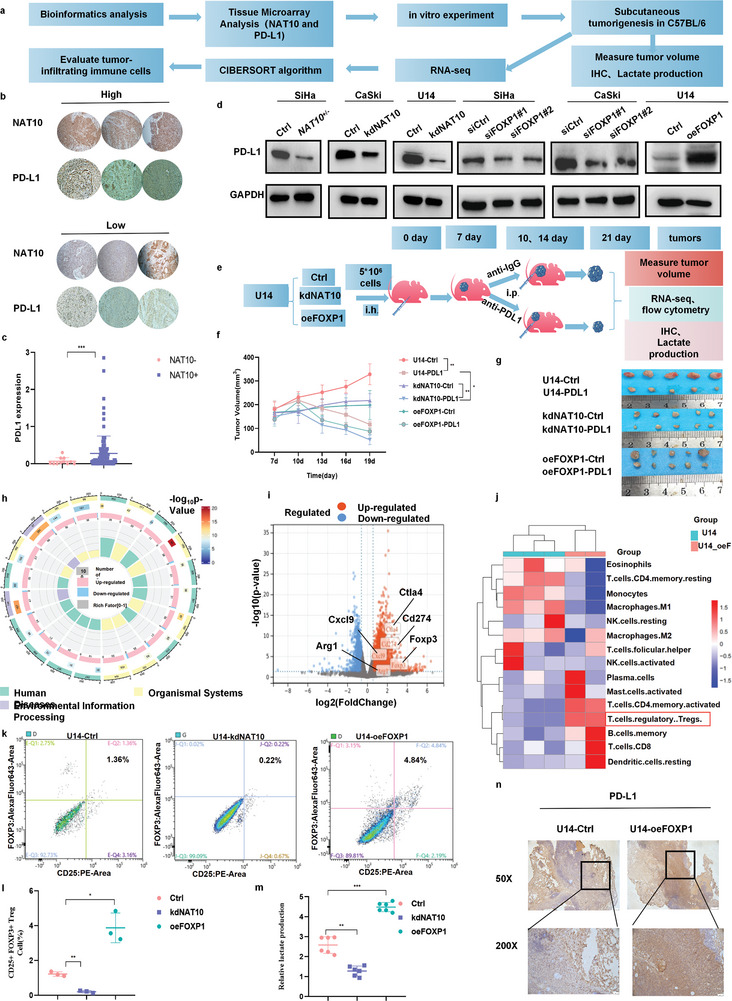
NAT10/ac4C/FOXP1 axis activity increases Treg infiltration in highly glycolytic tumors. a) Diagram showing the results of the correlation analysis and the mechanism linking NAT10, FOXP1, and CCa in the TME. b,c) Immunohistochemical analysis results showing that NAT10 upregulation was positively associated with increased PD‐L1 expression in CCa (n = 111). d) Western blot results confirming PD‐L1 downregulation in NAT10 knockdown and FOXP1‐deficient CCa cells compared with the corresponding control cells. e) Mouse models were successfully established in mice by subcutaneous injection of U14‐Ctrl, NAT10 knockdown U14 (U14‐kdNAT10) and FOXP1‐overexpressing U14 cells (U14‐oeFOXP1); f,g) The tumor size and tumor growth rate were calculated after treatment with IgG or a PD‐L1 inhibitor in the U14 NAT10 knockdown and FOXP1 overexpression groups versus the U14 control group of C57BL/6 mice (n = 5). h) GO analysis of the RNA‐seq results showing the differences in key functions and pathways between the groups of C57BL/6 mice bearing U14 control and U14 FOXP1‐overexpressing subcutaneous tumors. i) Volcano plot showing the upregulation of key DEGs, including CD274, CTLA4, CXCL9, ARG1, and FOXP3, in the U14‐oeFOXP1 group. j) The CIBERSORT algorithm was used to evaluate the differences in the proportions of immune cells in the TME between control and FOXP1 U14‐overexpressing subcutaneous tumors. k,l) Flow cytometric analysis was performed to evaluate and quantify the proportions of Tregs in the U14‐Ctrl, U14‐kdNAT10, and U14‐oeFOXP1 groups (n = 3). m) The level of lactate secreted in subcutaneous tumors derived from U14 cells with FOXP1 overexpression compared with those derived from U14 cells. n) Immunohistochemical staining demonstrating PD‐L1 expression in different tumors. The data are shown as the means ± SDs. *p<0.05; **p<0.01; *** *p*<0.001; **** *p*<0.0001; ns, nonsignificant.

As mentioned above, our study revealed that upregulation of NAT10 and FOXP1 promoted the proliferation, invasion, migration and glycolytic ability of CCa cells, suggesting that NAT10‐mediated acetylation of FOXP1 may be essential for immune evasion (**Figure** **8**a), as indicated by bioinformatics analysis (Figure 1g,h). To clarify the association between the NAT10/ac4C**/**FOXP1 axis and immune suppression in CCa (Figure 8a), immunohistochemical staining for PD‐L1 was performed and statistically analyzed in TMA cores, revealing a positive correlation between NAT10 and PD‐L1 expression in CCa patients (Figure 8b,c). Additionally, through Western blotting, we further validated the positive correlations among NAT10, FOXP1 and PD‐L1 in CCa cells (Figure 8d). To assess the effect of targeting the NAT10/ac4C/FOXP1 axis on tumor progression in vivo, we established a subcutaneous tumor model with U14‐kdNAT10 and U14‐oeFOXP1 cells in C57BL/6 mice and then treated these mice with an anti‐PD‐L1 antibody (2.5 mg kg^−1^ per day) on the 10th and 13th days after implantation. Interestingly, in mice treated with IgG as a control, NAT10 downregulation substantially suppressed CCa progression, while there was no discernible difference in tumor growth between the control and U14‐oeFOXP1 groups (Figure 8e,f). Moreover, to investigate the potential synergistic enhancement of anti‐PD‐L1 efficacy through combination treatment with NAT10 knockdown, we also monitored tumor growth after anti‐PD‐L1 antibody treatment in different groups in the model. Notably, the U14‐kdNAT10 group exhibited significantly slower tumor growth than the monotherapy groups (Figure 8f,g). Therefore, consistent with the role of the NAT10/ac4C/FOXP1 axis in facilitating the upregulation of immune checkpoint genes and subsequent immune evasion, inhibition of this axis resulted in PD‐L1 blockade‐induced tumor regression that correspondingly inhibited CCa progression. The combination therapy showed much better therapeutic efficacy than either monotherapy.

To evaluate NAT10/ac4C/FOXP1 axis‐induced immune infiltration in the TME of CCa, we analyzed the comprehensive molecular profile of NAT10‐induced FOXP1 overexpression under immunocompetent conditions by RNA‐seq analysis of samples from the U14‐Ctrl and U14‐oeFOXP1 models. Based on the RNA‐seq results, we performed GO enrichment analysis on the TME‐related DEGs, aiming to assess the alterations in the TME. As shown in Figure 8h, the DEGs were mainly enriched in metabolic process‐, biological regulation‐ and immune‐related GO terms, consistent with the previous results in this study. Furthermore, PD‐L1 upregulation was observed in tissues from mice in the U14‐oeFOXP1 group compared with tissues from mice in the control group, as shown in the volcano plot (Figure 8i); moreover, several key genes, including CTLA4, CXCL9, ARG1, and FOXP3, were upregulated in tissues from mice in the U14‐oeFOXP1 group, suggesting that FOXP1 overexpression may play an important role in checkpoint gene expression.

Moreover, we conducted correlation analysis of 22 types of tumor‐infiltrating immune cells (TIICs) with the CIBERSORT algorithm and found a distinct immunophenotypic profile in CCa. The results of differential expression and correlation analyses for FOXP1 and TIICs indicated that FOXP1 overexpression was positively correlated with the infiltration of Tregs and activated CD4+ T cells but negatively correlated with the infiltration of M1 macrophages, resting NK cells and resting CD4+ memory T cells (Figure 8j). Moreover, by analyzing TCGA data, we established positive correlations among FOXP3, TNFAIP6 and FOXP1 (Figure S2k,l, Supporting Information). Subsequently, we performed flow cytometric analysis and found that FOXP1 upregulation effectively promoted Treg infiltration within the TME, but NAT10 knockdown showed reverse trend (Figure 8k,l). Considered collectively, the differential expression and correlation analyses further substantiated the hypothesis that FOXP1 and NAT10 are intricately linked to the immunosuppressive TME in CCa tissues. Then, one month after tumor excision, lactate levels were measured within the TME and were found to be significantly higher in the U14‐oeFOXP1 group than in the U14‐Ctrl group, while lower in the U14‐kdNAT10 group (Figure 8m). Previous novel findings revealed that tumor‐derived lactic acid acts as a mediator of resistance to checkpoint blockade, thereby promoting Treg activity.^[^ ^34^ ^]^ Thus, an unexpected surge in PD‐1 expression occurred in Tregs exposed to a TME containing high levels of lactic acid.^[^ ^34^ ^]^ Notably, Tregs have been verified to take up lactate from the TME to support their survival and differentiation in vivo. Considering the crosstalk between metabolic dysregulation and immune cell infiltration, we detected and validated PD‐L1 expression in the different groups by immunohistochemistry, and the results showed an increasing trend of PD‐L1 expression within the TME in the U14‐oeFOXP1 group (Figure 8n). Taken together, our results showed the crosstalk between cancer metabolism and the immune response in CCa. Specifically, targeting the NAT10‐FOXP1 axis in CCa tumors could not only hamper tumor cell glycolysis but also decrease the infiltration of Treg populations into the TME, synergistically enhancing the efficacy of PD‐L1 blockade and ultimately preventing tumor progression.

## 3 Discussion

With the advent of cutting‐edge techniques such as transcriptome‐wide ac4C mapping, the biological significance, and indispensable role of mRNA modifications have recently been recognized.^[^ ^35^ ^]^ ac4C modification is predominantly enriched in coding sequences (CDSs) of mRNAs, and its enrichment at C bases at the wobble position in codons enhances translation efficiency through a transcriptome‐wide mechanism.^[^ ^36^ ^]^ Interestingly, the identification of NAT10 ushered in a new era in the understanding of the functions of ac4C modification in diverse biological processes, which are significantly associated with tumor aggressiveness and poor clinical outcomes.^[^ ^37^ ^]^ However, the functional importance of the ac4C writer NAT10 in CCa remains unexplored. Here, we investigated the localization of ac4C and the functional impact of its writer NAT10 by mapping ac4C‐modified RNAs in CCa cells, revealing that NAT10 plays an oncogenic role in CCa. Moreover, HOXC8 facilitated NAT10 transcription in CCa cells by binding to its promoter region, which facilitated the proliferation, migration and invasion of CCa cells in vitro and tumor progression in vivo. Using a multiomics screening strategy combining acRIP‐seq, Ribo‐seq and RNA‐seq data to delineate the mechanisms of NAT10 in CCa, we promisingly found that FOXP1 is the key downstream target of NAT10‐mediated ac4C modification.

NAT10, initially discovered in 2003, plays multifaceted functional roles in various cancers: it not only greatly facilitates cell proliferation in breast cancer but also is vitally involved in promoting melanogenesis and facilitating melanoma growth.^[^ ^38^, ^39^ ^]^ Bioinformatics analysis strongly indicates that NAT10 upregulation affects the prognosis of patients with diverse cancers and is closely related to tumor immune infiltration, contributing especially prominently to the regulation of TAMs, B cells, and exhausted T cells in hepatocellular carcinoma.^[^ ^37^ ^]^ However, the underlying functions and mechanisms of NAT10 in tumor progression and immunosuppression remain unclear. Herein, we elucidated that ac4C modification mediated by NAT10 increased FOXP1 expression in CCa, thereby leading to transcriptional upregulation of GLUT4 and KHK, in turn resulting in increased glycolysis. The consequent accumulation of lactate in the TME further promoted Treg infiltration. Impressively, NAT10 knockdown acted synergistically with PD‐L1 blockade therapy in vivo, leading to tumor regression by impairing tumor cell glycolysis, decreasing lactic acid production, attenuating immunosuppression and improving immunosurveillance (as shown in the Graphical Abstract).

FOXP1, a well‐known TF, performs dual functions in regulating the expression of distinct genes. FOXP1 deficiency is associated with poorer prognosis in breast cancer and promotes the development of lung carcinoma,^[^ ^40^ ^]^ whereas FOXP1 can serve as an oncogene leading to poor outcomes in large B‐cell lymphoma.^[^ ^41^ ^]^ Moreover, FOXP1 inhibits T follicular helper cell differentiation^[^ ^42^ ^]^ and is also essential for the optimal expression of FOXP3 during the onset of iTreg induction.^[^ ^43^ ^]^ This study revealed that FOXP1 is a key target of NAT10‐mediated ac4C modification in CCa and first elucidated its underlying functions and mechanisms in metabolic reprogramming and immunosuppression. Importantly, CUT&Tag sequencing revealed that the upregulated DEGs after FOXP1 overexpression were enriched in several key ontological terms related to cellular metabolic processes, especially glycolysis; in addition, FOXP1 was identified as a key TF activating GLUT4 and KHK expression to facilitate glycolysis in CCa cells. Notably, GLUT4 serves as a glucose transporter that accelerates glucose uptake,^[^ ^44^ ^]^ while KHK belongs to the ribokinase superfamily of kinases responsible for converting fructose into fructose‐1‐phosphate in the rate‐limiting first step of fructose metabolism; furthermore, the products of this reaction can participate in the glycolytic pathway.^[^ ^45^ ^]^

Abundant evidence emphasizes the propensity of cancer cells to exploit the RNA epitranscriptome to escape immune surveillance through the regulation of glycolytic metabolism, adapt to nutrient‐deficient circumstances and maintain their growth and proliferation.^[^ ^24^ ^]^ For instance, FTO‐mediated m^6^A modification regulates some TFs, such as c‐Jun and JunB, which subsequently dismantle the metabolic barrier, impeding T‐cell activation. The FTO inhibitor Dac51 could enhance the curative effect of PD‐L1 blockade therapy, as demonstrated in recent studies.^[^ ^46^ ^]^ Additionally, FTO inhibition has been shown to synergize with anti‐PD‐1 blockade and overcome resistance to immunotherapy in melanoma through the regulation of downstream targets, including PD‐1, CXCR4 and SOX10.^[^ ^47^ ^]^ FOXP3 participates in regulating the immune system as a specific marker for Treg development and function.^[^ ^48^ ^]^ Considering that FOXP family proteins exhibit functional similarities and that there are some protein–protein interactions closely associated with TME components, such as inflammatory cytokines, findings have confirmed that FOXP1 plays an indispensable role in Tregs by promoting FOXP3‐mediated regulation of gene expression.^[^ ^49^ ^]^ The surprising finding that PD‐1 blockade synergizes with lactic acid inhibition to enhance Treg suppression and hinder antitumor immunity^[^ ^27^ ^]^ constitutes important evidence in this study. The findings of the current study reveal a mechanism by which NAT10‐mediated FOXP1 overexpression induces increased glycolysis in CCa cells and enhances Treg infiltration into the TME, leading to immune evasion in CCa, and further lay a solid foundation for the development of NAT10 inhibitors as novel therapeutic agents.

## 4 Conclusion

In conclusion, we provided compelling in vitro and in vivo evidence revealing an oncogenic role of NAT10 in CCa progression and determined that as an essential epitranscriptomic regulator, NAT10‐mediated ac4C modification in tumor cells upregulates the TF FOXP1, which increases Treg infiltration and promotes CCa immunosuppression by reprogramming glycolytic metabolism. Furthermore, NAT10 knockdown profoundly improved the efficacy of PD‐L1 blockade therapy by impairing tumor cell glycolysis, reducing lactic acid production, and enhancing immunosurveillance within the TME, ultimately resulting in tumor regression. This elucidation of NAT10‐mediated ac4C modification is expected to significantly contribute to further investigation of CCa and facilitate the development of promising therapeutic strategies.

## 5 Experimental Section

### Cell Culture

The human epithelial foreskin fibroblast line (HFF‐1) used as noncancerous control cells^[^ ^50^ ^]^ and the human CCa cell lines SiHa and CaSki were purchased from the Cell Bank of the Chinese Academy of Sciences and were cultured in DMEM (Gibco, US) and RPMI 1640 medium (Gibco, US) in a humidified atmosphere with 5% CO_2_ at 37 °C according to the corresponding guidelines. Furthermore, U14 cervical carcinoma cells were purchased from Boster Company and cultured in DMEM (Gibco, US). All cell lines were tested for authenticity using short tandem repeat genotyping and were routinely tested for mycoplasma contamination.

### Animal Studies

Female BALB/c nude mice and approximately 6‐ to 8‐week‐old C57BL/6 mice were purchased from GemPharmatech Company Animal Center (Foshan, China) and raised under pathogen‐free conditions. The procedures for animal care and use were approved by the Ethics Committee of the Shenzhen Hospital of Southern Medical University (NO.2022‐0099). For the subcutaneous xenograft model, cells were prepared and injected subcutaneously into the right dorsal surface of nude mice, and the luciferase signal intensity was measured weekly and calculated the tumor volume (V = (tumor width (W)^2^ × tumor length (L))/2) every three days after tumor formation.

In addition, U14‐Ctrl or U14‐oeFOXP1 cells were injected into the right subaxillary region of C57BL/6 mice. The luciferase signal intensity was measured weekly to verify successful tumor growth and then excised the tumors for flow cytometry and RNA‐seq analysis on the 7th day. For anti‐PD‐L1 treatment, 5 × 10^6^ U14‐Ctrl, U14‐kdNAT10 or U14‐oeFOXP1 tumor cells were subcutaneously injected into the right dorsal surface of C57BL/6 mice. On days 10 and 14, 5 mg of an anti‐PD‐L1 antibody (ichorbio, ICH1086, clone: 10F.9G2) or mouse immunoglobulin (anti‐mouse IgG) was diluted in PBS and injected intraperitoneally into the tumor‐bearing mice. The size of each tumor was recorded every three days to generate growth curves.

### Plasmid Construction, Lentivirus Production, and Cell Transduction

The shRNA plasmids used for lentivirus‐mediated interference with FOXP1 expression were synthesized, annealed and cloned by Shanghai Genechem Company (Guangzhou, China). The related shRNA sequences were shown in Table S2b (Supporting Information). The NAT10‐wt (NAT10‐Flag) and NAT10‐mut (del 558‐753aa) expression plasmids were constructed in the GV657 vector (GeneChem, Shanghai). All siRNAs were designed and synthesized by RiboBio (Guangzhou, China), and the detailed siRNA sequences were listed in Table S2b (Supporting Information). To generate a cell line with stable *NAT10* heterozygous knockdown (*NAT10^+/‐^* SiHa), two gRNA sets were designed and inserted into the vector LV‐U6>gRNA‐A1‐U6>gRNA‐A2‐CMV>P2A/Hygro (Cyagen, Guangzhou) using the Cas9 gene editing system by a team from Haixing Biosciences (Guangzhou, China). The sequences of the NAT10 sgRNAs were as follows: sgRNA‐1, ACTGCACGGATAGCAAGTGG; sgRNA‐2, CCAAAGGAAGATAATGCACAA. ≈10 single cell‐derived clones were established and confirmed via PCR. The *NAT10* heterozygous knockdown (*NAT10* ^+/−^) SiHa cell lines and control cell lines were further confirmed via Western blot analysis of NAT10 expression.

### Tissue microarray (TMA) Construction

The CCa TMAs were constructed by Shanghai OUTDO Biotech Co. (Shanghai, China) and included samples from a cohort of 144 patients (Table S1, Supporting Information). The tissue samples were blocked with a primary antibody (anti‐NAT10, ab194297) and finally scored independently by two pathologists in a blinded manner by calculation of a semiquantitative immunoreactivity score (IRS) in the cohort; an IRS of <4 was considered to indicate low NAT10 expression, and an IRS of >4 was considered to indicate high NAT10 expression. In addition, the grading of NAT10 and FOXP1 staining in 20 cases of cervical cancer by immunohistochemistry was displayed in Table S3 (Supporting Information).

### Immunohistochemistry and HE Staining

All tissues were fixed, embedded, sliced into sections, dewaxed with xylene, hydrated, subjected to antigen retrieval and incubated with antibodies overnight. The next day, the sections were incubated with secondary antibodies (ZSGB‐BIO, PV‐6000) and then stained with DAB reagent and hematoxylin. Finally, fluorescence images were acquired, reviewed and analyzed. The primary antibodies were as follows: anti‐FOXP1 (1:100, Cell Signaling Technology, 4402S), anti‐NAT10 (1:500, Abcam, ab194297), anti‐GLUT4 (1:100, Proteintech), and anti‐KHK (1:100, Proteintech, 15681‐1‐AP).

### Dual‐Luciferase Reporter Assay

All plasmids were designed and constructed by Obio Technology (Shanghai). The plasmids and Renilla luciferase reporter vector were cotransfected into cells with Lipofectamine 3000 (Thermo Fisher) for 36 h. With the dual‐luciferase reporter assay system (Promega), firefly luciferase and Renilla luciferase activities were measured in each well, and the ratio of firefly luciferase activity to Renilla luciferase activity was finally calculated.

### Cell Proliferation Assay

In the Cell Counting Kit‐8 (CCK‐8) assay, after treatment with CCK‐8 reagent (Meilunbio, China) for 2 h, cell proliferation ability was evaluated based on the absorbance at 450 nm. To complement the CCK‐8 assay, a colony formation assay was performed. Cells were plated at a density of 1000 cells per well in 6‐well plates and incubated for 2 weeks, and the colonies were then fixed with methanol for 30 min and stained with crystal violet (Biosharp, China) for 10 min.

### Transwell Assays

Transwell inserts containing an 8‐µm PC membrane (Corning) was used to perform Transwell assays to evaluate the migration and invasion abilities of CCa cells. Cells were suspended in medium without FBS and seeded in the upper chamber at a density of 5 × 10^5^ cells per well, while 600 µl of culture medium containing 10% FBS was placed in the lower chamber. Then, after 12 h of incubation, the cells on the lower surface of the membrane were fixed, stained and imaged.

### Immunofluorescence (IF) Assay

Cells were incubated first with an anti‐NAT10 antibody (Abcam, ab194297) at 4°C overnight and then with the corresponding Alexa Fluor‐labeled secondary antibody for 1 h at room temperature. Finally, the cells were incubated with DAPI (Beyotime, Shanghai, China) for 5 min and imaged.

### Glycolysis Assay

Lactate and adenosine triphosphate (ATP) production were quantified using a lactic acid assay kit (A019‐2‐1, Jiancheng Bioengineering Institute, China) and an ATP assay kit (S0027, Beyotime, China), respectively, according to the manufacturers’ instructions. In addition, CCa cells were seeded and incubated overnight, and the medium was then refreshed with glucose‐free DMEM supplemented with 10% dialyzed FBS. Twenty‐four hours later, the cells were treated with 2‐NBDG (20 µM; APExBIO) for 30 min, and glucose uptake was quantified. The number of cells per well was 2*10^4^, and the ECAR was measured with an Agilent Seahorse XFe96 Analyzer using the Seahorse XF Glycolytic Rate Assay Kit (Agilent). All results were normalized and analyzed using Wave software (Agilent).

### ac4C Acetylated RNA Immunoprecipitation followed by Sequencing (acRIP‐seq) and acRIP‐qPCR

RNA fragments were incubated with an anti‐ac4C antibody (Abcam, ab252215) or IgG (Abcam, ab172730), and ac4C‐modified RNA was then eluted with N‐acetylcytidine sodium salt for ac4C enrichment analysis by qPCR. The primer sequences used for acRIP‐qPCR were shown in Table S2a (Supporting Information). The libraries were generated and sequenced according to the manufacturer's protocol (Epibiotek, China).

### Ribosome Profiling Sequencing (Ribo‐seq)

Both SiHa and *NAT10* ^+/‐^ SiHa CCa cells were pretreated with 100 µg ml^−1^ cycloheximide (CHX), and RNA fragments protected by ribosomes were detected by second‐generation sequencing according to the manufacturer's protocol (Epibiotek, China).

### Assay for Transposase‐Accessible Chromatin Followed by Sequencing (ATAC‐seq)

In brief, H8 cells (immortalized normal human cervical epithelial cells), SiHa cells, and HFF‐1 (human epithelial foreskin fibroblasts) control cells (5 × 10^5^ cells each), were processed according to a previously published protocol.^[^ ^51^ ^]^ The final samples were purified, and sequencing was performed using an Illumina NovaSeq 6000 system. This service was provided by Guangzhou Saicheng Biotechnology.

### Cleavage Under Targets and Tagmentation (CUT&Tag) and ChIP‒qPCR

CUT&Tag was performed according to published methods with minor modifications^[^ ^52^ ^]^ by Jiayin Biomedical Technology Company (Shanghai, China). A ChIP assay kit (Millipore, Bedford, MA) was utilized according to the manufacturer's instructions. The sequences of the primers used for ChIP‒qPCR were also listed in Table S2a (Supporting Information).

### Immune Cell Profiling

Differences in the immune cell composition in tumor tissues were estimated with the CIBERSORT algorithm (https://cibersortx.stanford.edu/) to assess the infiltration of 22 immune cells in the U14‐Ctrl and U14‐oeFOXP1 groups. Statistical significance was defined as a *P* value < 0.05.

### Flow Cytometry

Tissues were digested, filtered, and stained with antibodies. Finally, the True‐Nuclear™ Transcription Factor Buffer Set (424401, BioLegend) was used for fixation and permeabilization of cells for intracellular staining of FOXP3 with anti‐mouse antibody. Finally, the cells in the TME were examined by flow cytometry. The following antibodies were purchased from BioLegend: anti‐mouse CD45 (103114), anti‐mouse CD4 (100451), anti‐mouse CD25 (102007), and anti‐mouse FOXP3 (126407).

### Western Blot Analysis

Based on previous reports, the following antibodies were used in this study: anti‐NAT10 (Abcam, ab194297), anti‐FOXP1 (Cell Signaling Technology, 4402S), anti‐HOXC8 (Abnova, H00003224‐M02), anti‐FLAG (Sigma, F1804), anti‐GLUT4 (Proteintech, 66846‐1‐Ig), anti‐KHK (Proteintech, 15681‐1‐AP), anti‐β‐tubulin (Yeasen, 30301ES40) and anti‐GAPDH (Yeasen, 30201ES20).

### Quantitative Reverse Transcription PCR (qRT‒PCR)

RT‒PCR was performed with the Hifair® II 1st Strand cDNA Synthesis Kit for qPCR (Yeasen, 11121ES60) and the SYBR Green PCR Kit (Yeasen, 111184ES03) on an Applied Biosystems 7500 sequence detection system with triplicate reactions. The primers used were listed in Table S2a (Supporting Information).

### mRNA Stability Assay

mRNA stability was evaluated by incubating cells with 5 µg ml^‐1^ actinomycin D (ActD, Catalog #A9415, Sigma, US) and isolating RNA for RT–qPCR to calculate the half‐life (t1/2) of FOXP1 mRNA.

### Quantification and Statistical Analysis

All results were expressed as the means ± SDs, and statistical analysis was performed using GraphPad Prism 7.0; differences were considered statistically significant when the *P* value was <0.05. The significance of differences was evaluated using two‐tailed unpaired Student's t test or one‐way ANOVA as appropriate. Moreover, the relationships between NAT10 expression and the histological subtype and HPV infection status of patients with CCa were analyzed using the Pearson chi‐square test. Statistical significance was denoted by * (*p* < 0.05), ** (*p* < 0.01), *** (*p* < 0.001) and **** (*p* < 0.0001) in the figures.

### Ethical Statement

The CCa TMAs were constructed by Shanghai OUTDO Biotech Co. (Shanghai, China) and the study was approved by the Institutional Review Board of Shanghai OUTDO Biotech Co. (SHYJS‐CP‐1801011). In addition, the 20 cases of CCa tissues and 20 cases of normal cervical tissues were obtained during surgery. The written informed consent was obtained from all patients, and the study was approved by the Institutional Review Board of Affiliated Tumor Hospital of Xinjiang Medical University (G‐201442).

This study was supervised by the Ethics Committee of Shenzhen Hospital of Southern Medical University to ensure that the research work carried out is in line with the relevant provisions of medical ethics.

## Conflict of Interest

The authors declare no conflict of interest.

## Author Contributions

X.C., Y.H. and Y.L. contributed equally to this work. Conceived and designed the experiments: X.G. and J.Z. Funding acquisition: X.G., X.L., Y.H., and Y.L. Performed the experiments: X.C., S.Z., K.A., and Y.Y. Analyzed the data: T.C., M.Y., H.H., and J.C. Contributed reagents/materials/analysis tools: X.L., Y.H., Y.L., M.Y., and A.L. Wrote the paper: X.C. and Y.H. Revised the paper: X.C., X.G., T.C., and X.L. All authors read and approved the final version of the submitted manuscript.

## Supporting information

Supporting Information

## Data Availability

The data that support the findings of this study are available from the corresponding author upon reasonable request.
