# Supplementary material for: NAT10/ac4C/FOXP1 Promotes Malignant Progression and Facilitates Immunosuppression by Reprogramming Glycolytic Metabolism in Cervical Cancer
Source: Adv Sci (Weinh). 2023 Oct 11;10(32):2302705. doi: 10.1002/advs.202302705 (PMC10646273; doi:10.1002/advs.202302705)
Supplement: Supplementary file 1 — Supporting Information [file ADVS-10-2302705-s001.pdf]

ADVANCED SCIENCE
Open Access

Supporting Information

for *Adv. Sci.*, DOI 10.1002/advs.202302705

NAT10/ac4C/FOXP1 Promotes Malignant Progression and Facilitates Immunosuppression by Reprogramming Glycolytic Metabolism in Cervical Cancer

Xiaona Chen, Yi Hao, Yong Liu, Sheng Zhong, Yuehua You, Keyi Ao, Tuotuo Chong, Xiaomin Luo, Minuo Yin, Ming Ye, Hui He, Anwei Lu, Jianjun Chen, Xin Li, Jian Zhang* and Xia Guo**

NAT10/ac4C/FOXP1 promotes malignant progression and facilitates immunosuppression by reprogramming glycolytic metabolism in cervical cancer

Xiaona Chen[1,2#], Yi Hao[3#], Yong Liu[4#], Sheng Zhong[1,2], Yuehua You[5], Keyi Ao[1,2], Tuotuo Chong[1,2], Xiaomin Luo[1,2], Minuo Yin[6], Ming Ye[7], Hui He[8], Anwei Lu[6], Jianjun Chen[9], Xin Li [1,2*], Jian Zhang[10*], Xia Guo[1,2*]

Supplementary materials

Supplementary Table 1 NAT10 protein expression in the tissue microarray and its relationship with patients' clinicopathological characteristics.

		NAT10-low （%）	NAT10-high （%）	P 值
**Age**				
<50	104	75（66.2%）	29（33.8%）	*P*=0.403
≥50	40	26（62.2%）	14（37.8%）	
**Pathological type**				
Normal	32	30（93.8%）	2（6.3%）	
CIN3	17	8（47.1%）	9（52.9%）	*P*=0.001
Carcinoma	95	63（66.3%）	32（33.7%）	
**Clinical stage（TMN）**				
Ⅰ	64	39（60.9%）	25（39.1%）	
Ⅱ	27	16（59.3%）	11（40.7%）	*P*=0.397
Ⅲ	21	16（76.2%）	5（23.8%）	
**HPV infection**				
Yes	91	61（67.0%）	30（33.0%）	*P*=0.023
No	47	40（85.1%）	7（14.9%）	

Supplementary Table 2a The primer sequences for qRT-PCR, ChIP-qPCR and acRIP-qPCR.

Primers	Sequences
GAPDH-Forward	CGGAGTCAACGGATTTGGTCGTAT
GAPDH-Reverse	AGCCTTCTCCATGGTGGTGAAGAC
HOXC8-Forward	ACCGGCCTATTACGACTGC
HOXC8-Reverse	TGCTGGTAGCCTGAGTTGGA
NAT10-Forward	GCCTCTTGTAAGAAGTGTCTCG
NAT10-Reverse	TCTTTTCAGAGATGCCCTCGAT
FOXP1-acRIP-F	GGAAATCCCACTCTGGGCAA
FOXP1-acRIP-R	ACTGTGGTTGGCTGTTGTCA
FOXP1-Forward	CTCAAGGCATGATTCCAACA
FOXP1-Reverse	GCATGTGGGTTCATTATTAAGGA
KHK-Forward	CTAAGGAGGACTCGGAGATAAGG
KHK-Reverse	CATTGAGCCCATGAAGGCAC
GLUT4-Forward	CGTCTCCATTGTGGCCATCT
GLUT4-Reverse	CCCATAGCCTCCGCAACATA
GLUT4-chip-Forward	TCCGAAGCCCGTTTTCTGAG
GLUT4-chip-Reverse	GTAACCCGGGGCTGCTATTT

Supplementary Table 2b The sequences of siRNAs and shRNAs.

siRNAs	Sequences
siFOXP1#1	CTGGTTCACACGAATGTTT
siFOXP1#2	CTCAGTCCACACTCCCAAA
siHOXC8#1	GAAGGACAAGGCCACTTAA
siHOXC8#2	GCAGTGGACGGCAAACTTA
NAT10-gRNA#1	ACTGCACGGATAGCAAGTGG
NAT10-gRNA#2	CCAAGGAAGATAATGCACAA
FOXP1-RNAi	gtGCGAAGATTTCCAATCATT

Supplementary Table S3 The grading of NAT10 and FOXP1 staining in 20 cases of cervical cancer by immunohistochemistry

Cases	grading of NAT10	grading of FOXP1	Cases	grading of NAT10	grading of FOXP1
1	+++	++	11	+/-	+/-
2	+	+	12	+	+
3	+	-	13	+++	-
4	++	+	14	++	++
5	+	-	15	+++	+
6	+	++	16	+	+
7	+	-	17	+++	++
8	++	++	18	+++	+++
9	+++	++	19	+++	+++
10	++	++	20	++	++

The NAT10 and FOXP1 staining <2+ intensity or ⩾ 2+ intensity were respectively identified as low expression group and high expression group; *P* value was calculated by Fisher exact test and $p < 0.05$ was considered statistically significant.

Figure S1

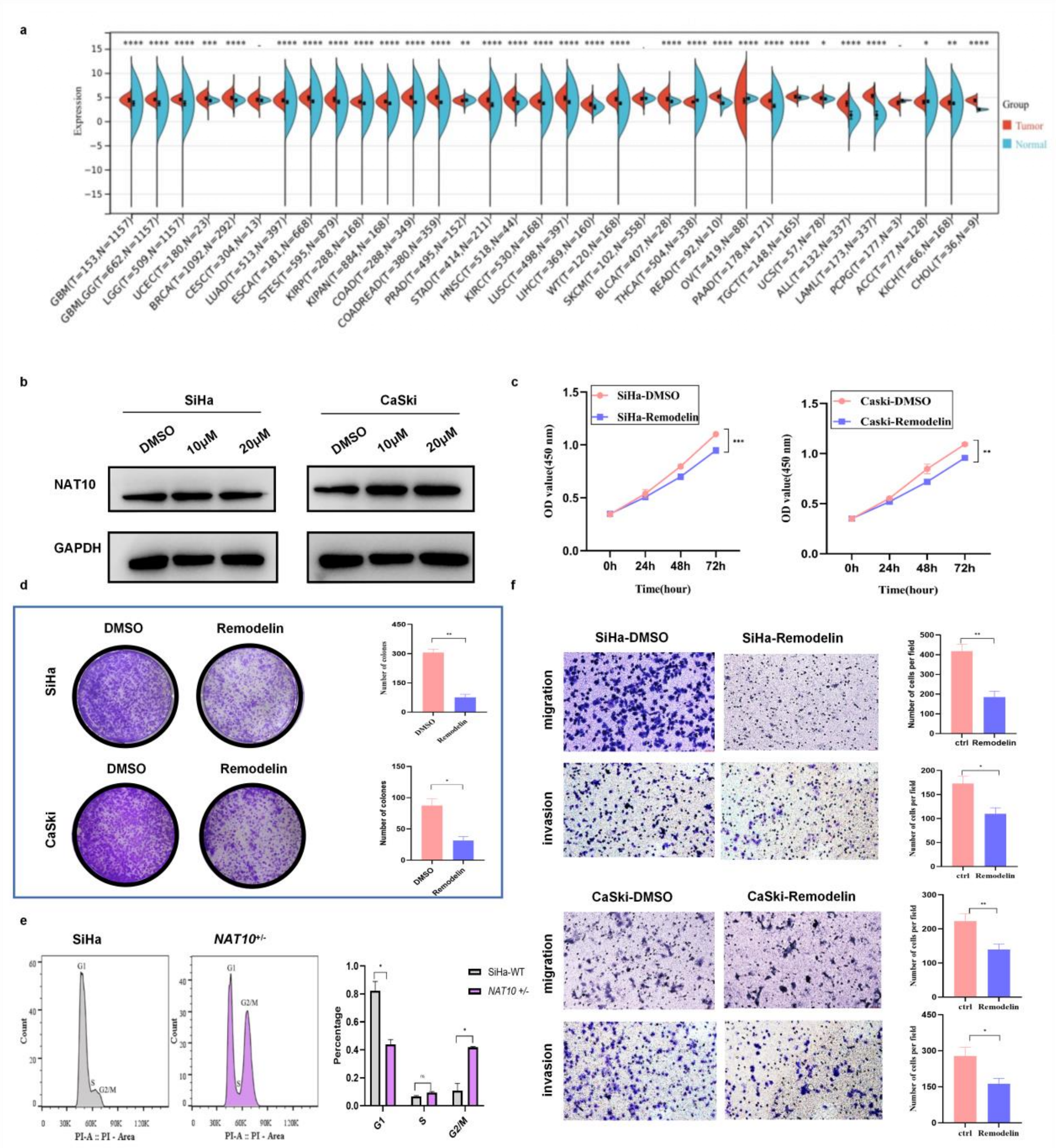

(a) TCGA database analysis showed the expression level of NAT10 in various cancers (tumour) and adjacent normal tissues (normal) through Sanger Box. (b) Western blot analysis revealed the expression of NAT10 when cells were treated with Remodelin. (c) Inhibition of NAT10 impaired cell viability in cervical cancer by the CCK8 assay. (d) Treatment with Remodelin reduced the ability of colony formation in CCa cells. (e) The cell cycle was

analysed by flow cytometry in the SiHa cells. (f) Treatment with Remodelin inhibited the migration or invasion ability of CCa cells.

Figure S2

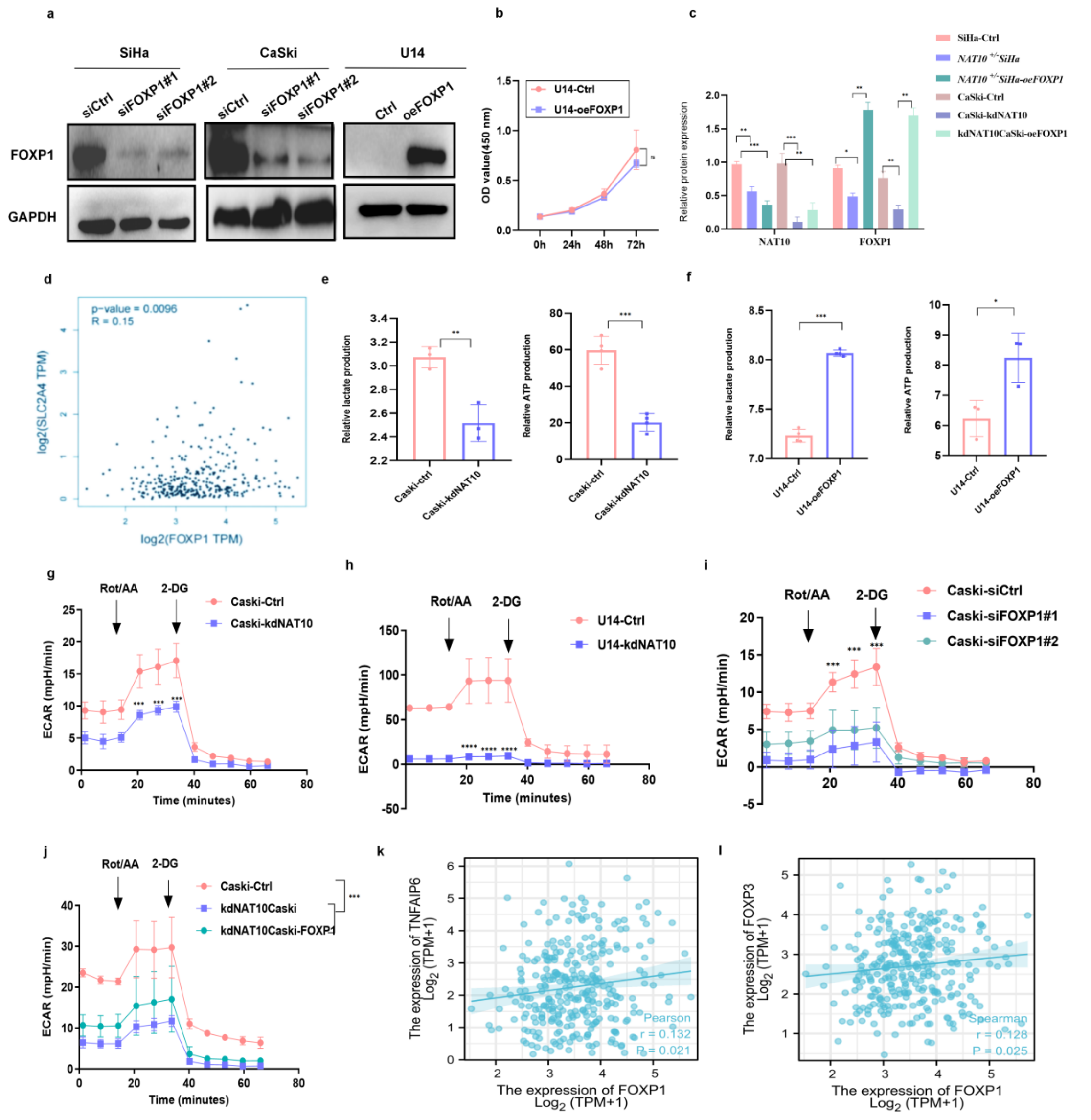

(a) Verification of the knockdown efficiency of siRNAs targeting FOXP1 in CCa cells and the successful establishment of U14 cell lines with FOXP1 overexpression. (b) CCK8 assay results showing the proliferation ability of U14 and U14-oeFOXP1 cells. (c) The quantification of westernblot results among six groups. (d) The correlation between FOXP1 and GLUT4 (SLC2A4) expression was evaluated using GEPIA. (e-f) Knockdown of NAT10 reduced lactate and ATP production in CaSki cells (e), while overexpressing FOXP1 increased lactate and ATP production in U14 cells (f). (g-j) The ECAR profile was monitored

in NAT10 knockdown CaSki cells (g-h), FOXP1 knockdown CaSki cells (i) and NAT10 knockdown CaSki cells with FOXP1 overexpression(j). (k-l) The positive correlations among FOXP1, TNFAIP6 (k) and FOXP3 (l) expression were evaluated using Xiantao Xueshu.
